# Artificial Chlorella Biohybrids for Alleviation of Cartilage Degeneration in Osteoarthritis

**DOI:** 10.1002/EXP.20250125

**Published:** 2026-07-10

**Authors:** Yongyun Chang, Keyu Kong, Minghao Jin, Shasha Liu, Yangzi Yang, Yingchun Zhu, Hua Qiao, Wenxuan Fan, Xinru Wu, Jingwei Zhang, Yansong Qi, Yongsheng Xu, Xiang Feng, Pingcuo Nima, Piting Ji, Tariel Mamuladze, Andrei A. Korytkin, Huiwu Li, Jingke Fu, Zanjing Zhai

**Affiliations:** ^1^ Shanghai Key Laboratory of Orthopedic Implants Department of Orthopedics Ninth People's Hospital Shanghai Jiao Tong University School of Medicine Shanghai China; ^2^ Shanghai Engineering Research Center of Innovative Orthopaedic Instruments and Personalized Medicine Clinical and Translational Research Center for 3D Printing Technology Shanghai China; ^3^ Department of Rehabilitation Medicine Shanghai Ninth People's Hospital Shanghai Jiao Tong University School of Medicine Shanghai China; ^4^ Department of Orthopedic Surgery Spine Center Changzheng Hospital Navy Medical University Shanghai China; ^5^ Key Laboratory of Inorganic Coating Materials Shanghai Institute of Ceramics Chinese Academy of Sciences Shanghai P. R. China; ^6^ Department of Orthopedics Inner Mongolia People's Hospital Hohhot China; ^7^ Department of Orthopedics People's Hospital of Shigatse City Xizang Autonomous Region Shigatse City China; ^8^ Novosibirsk Scientific Research Institute of Traumatology and Orthopedics. Ya.L. Tsivyan: Novosibirsk Novosibirsk Oblast Russia

**Keywords:** cartilage degeneration, chondrocytes, mitophagy, glycolysis, osteoarthritis

## Abstract

Osteoarthritis (OA), one of the most common chronic joint diseases, is characterised by cartilage imbalance and disruption of the cartilage extracellular matrix. In this study, we observed impaired mitophagy and glucose metabolism disorders in OA chondrocytes, which exacerbated cartilage degeneration. And we unveil *Chlorella*, a natural microorganism that appeared more than two billion years ago, as an efficient regulator of both mitophagy and glucose metabolism in OA chondrocytes. *Chlorella* activates mitophagy and rescues mitochondrial function. More importantly, *Chlorella* inhibits glycolysis and reprograms glucose metabolism in chondrocytes, leading to the remodelling of chondrocyte homeostasis and alleviation of cartilage degeneration both in vitro and in vivo. As a proof of concept, we constructed a photothermal *Chlorella* biohybrid (Ch@P) that induced articular thermal stimulation under near‐infrared irradiation and significantly strengthened the protection against cartilage degeneration in DMM mouse models of OA. Ch@P activates the AMPK‐Sirt1 signalling pathway to restore mitochondrial homeostasis and energy metabolism in chondrocytes. Furthermore, HSP70 is also activated to regulate chondrocyte homeostasis due to articular thermal stimulation. Our study unveils the activity of natural *Chlorella* in chondrocyte homeostasis by activating mitophagy and reprogramming glucose metabolism and identifies an artificial Chlorella biohybrid as a promising therapeutic option for OA treatment.

## Graphical abstract

Artificial *Chlorella* biohybrids (Ch@P) alleviate cartilage degeneration and protect against OA progression by activating mitophagy and reprogramming glucose metabolism in chondrocytes, as well as inducing articular thermal stimulation under near infrared irradiation.

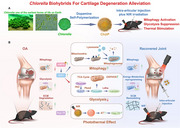

## 1 Introduction

Osteoarthritis (OA), the most prevalent chronic degenerative joint disease, has emerged as a leading cause of dysfunction and disability worldwide, particularly in older populations [1, 2]. OA is characterised by multiple morbidities, including articular cartilage degeneration, synovial inflammation, subchondral osteosclerosis and osteophyte formation [3, 4]. Progressive degeneration of articular cartilage is a major hallmark of OA [5]. Articular cartilage is a physiologically poor self‐repairing avascular and aneural tissue comprising chondrocytes and an extracellular matrix (ECM) [6]. Owing to the difficulty of cartilage recovery after damage, current therapies, including surgical interventions and the use of analgesic drugs, are palliative but not curative [7, 8, 9]. Considering the large number of people affected by OA, it is critical to explore its pathogenesis and develop more effective therapeutic strategies.

Mitochondria are important organelles that are responsible for cellular metabolism and energy production in eukaryotic cells. They play critical roles in the maintenance of chondrocyte metabolic homeostasis [10, 11]. Moreover, they are involved in crucial cellular processes, such as heat production, response to reactive oxygen species (ROS) and regulation of apoptosis and autophagy [12, 13, 14]. Emerging findings implicate mitochondrial dysfunction as a key mechanism in osteoarthritis, where it promotes the degradation of the cartilage matrix by triggering chondrocyte apoptosis and upregulating the activity of matrix‐degrading enzymes [15, 16, 17, 18]. Notably, mitochondrial dysfunction is often accompanied by reduced ATP synthesis, which is closely associated with alterations in energy metabolism patterns in OA [19]. Chondrocytes use glucose metabolism, including glycolysis and oxidative phosphorylation (OXPHOS) pathways, to produce ATP as their energy source [20]. However, in an OA‐inflammatory environment, chondrocytes experience a pathological change in metabolic homeostasis, marked by increased glycolysis, mitochondrial dysfunction and chondrosenescence [21, 22, 23, 24]. Glucose metabolism disorders can lead to chondrocyte hypertrophy and the breakdown of ECM, thereby contributing to the progression of OA. Hence, rescuing mitochondrial function and reprogramming glucose metabolism in chondrocytes could provide attractive therapeutic strategies for the treatment of OA.

In this study, we unveiled that single‐cell *Chlorella vulgaris* (abbreviated as *Chlorella* thereafter), one of the earliest forms of life on Earth that appeared more than 2 billion years ago, could be biohybridised into an efficient therapeutic option to rescue mitochondrial function and reprogram glucose metabolism of chondrocytes, alleviating cartilage degeneration in OA. *Chlorella* is a spherical unicellular green microalga found in eutrophic freshwater. It comprises a variety of bioactive constituents, such as polysaccharides, proteins and lipids, exhibiting potential antioxidant, anti‐inflammatory and immunomodulatory activities [25, 26]. *Chlorella* or *Chlorella* extracts have been shown to modulate immune responses, inhibit bacteria and exhibit significant anti‐cancer effects against a variety of cancer types [27, 28]. However, no studies have explored the use of *Chlorella* or *Chlorella*‐based biological systems for OA treatment.

**FIGURE 1:**
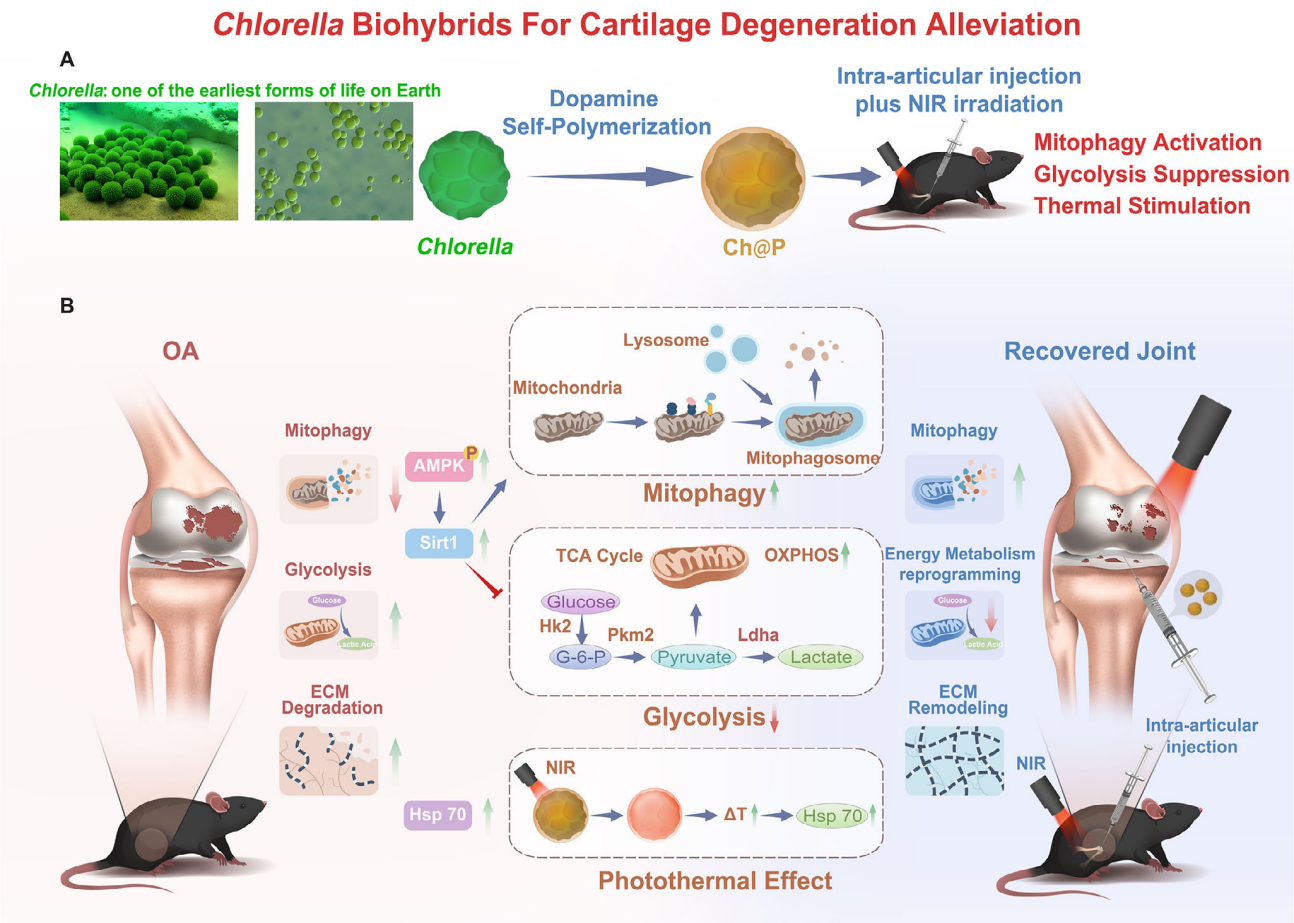
Schematic illustration of artificial *Chlorella* biohybrids (Ch@P) to alleviate cartilage degeneration and protect against OA progression by activating mitophagy and reprogramming glucose metabolism in chondrocytes, as well as inducing articular thermal stimulation under near infrared (NIR) irradiation. (A) The diagram shows the coating of polydopamine (PDA) onto natural *Chlorella* by dopamine oxidation and self‐polymerisation to generate Ch@P. (B) Ch@P promoted the expression of phosphorylated AMPK and its downstream Sirt1, leading to the alleviation of chondrocyte extracellular matrix (ECM) degradation by activating mitophagy and inhibiting glycolysis. Moreover, Ch@P activated Hsp70 in response to photothermal effect in chondrocytes under NIR stimulation, further strengthening the amelioration of OA in DMM mouse models.

For the first time, the findings of this study showed that *Chlorella* was able to rescue the mitochondrial function of chondrocytes by activating mitochondrial autophagy (mitophagy). More importantly, *Chlorella* could reprogram glucose metabolism in chondrocytes, reduce glycolysis and enhance OXPHOS. Consequently, *Chlorella* promoted chondrocyte ECM anabolism, inhibited catabolism in vitro, protected against cartilage degeneration, relieved pain sensitivity and ameliorated abnormal gait in OA mouse models. Moderate thermal stimulation of articular cartilage has been shown to suppress cartilage degeneration and thermotherapy has been widely used in clinical practice for the treatment of OA because of its effectiveness in relieving pain, promoting fluidity of joint synovial fluid and improving blood flow in muscles [29]. Natural *Chlorella* was further engineered using polydopamine (PDA) to construct a photothermal *Chlorella* biohybrid (Ch@P). The Ch@P biohybrid not only maintained the previously mentioned biological activity of *Chlorella* but also strongly alleviated cartilage degeneration both in vitro and in vivo due to mild thermal stimulation under near‐infrared (NIR) irradiation. Mechanistically, we found that Ch@P biohybrids continuously activated the adenosine monophosphate (AMP)‐activated protein kinase (AMPK)‐Sirtuin 1 (Sirt1) signalling pathway to regulate mitophagy and glycolysis in chondrocytes. Notably, Ch@P further activated heat shock protein 70 (HSP70), a key regulator of chondrocyte homeostasis and a favourable modulator of chondrocyte function (Figure 1). Our findings shed light on the therapeutic activity of natural *Chlorella* in restoring mitochondrial function and reprogramming glucose metabolism in chondrocytes and represent a major breakthrough in the treatment of OA.

## 2 Results

### 2.1 Mitophagy Impairment and Glycolysis Enhancement in OA Chondrocytes

**FIGURE 2:**
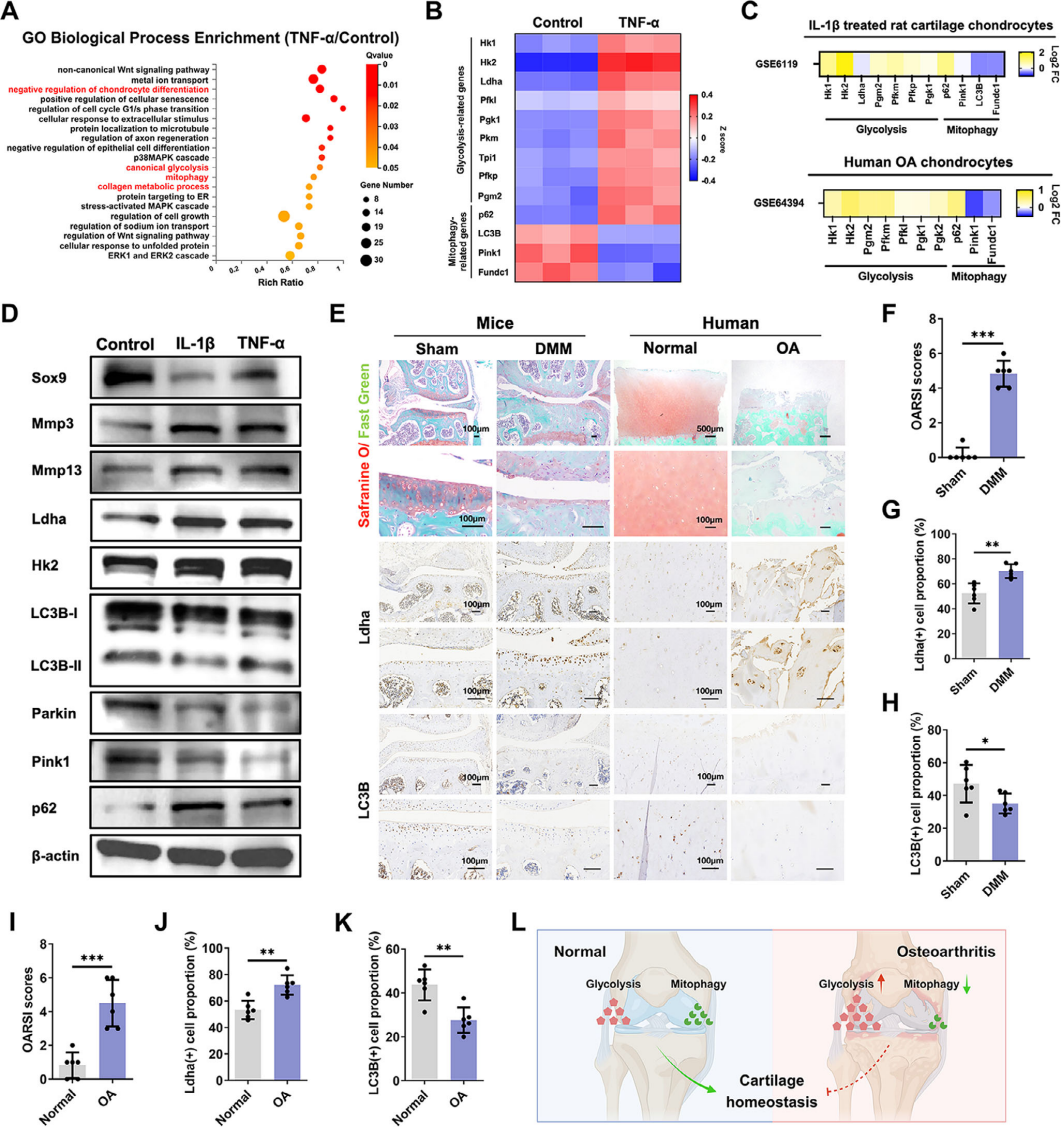
Mitophagy is impaired and glycolysis is enhanced in OA chondrocytes. (A) Gene ontology (GO) enrichment analysis of differential expressed genes (DEGs) in chondrocytes, with or without TNF‐α treatment (10 ng/mL). (B) The heatmap of differentially expressed mitophagy‐ and glycolysis‐related genes in chondrocytes, with or without TNF‐α treatment. (C) The heatmap of mitophagy‐ and glycolysis‐related genes in different GEO datasets. (D) Inflammatory factor treatment decreased the expression of ECM anabolism and mitophagy‐related proteins and increased ECM catabolism and glycolysis‐related proteins. (E‐K) Safranin O/fast green staining and immunohistochemical (IHC) staining of articular cartilage in OA mice and patients and the corresponding quantitative analyses. (L) The imbalance of mitophagy and glycolysis in OA chondrocytes. Data are presented as means ± SD derived from six replicates. *p < 0.05, **p < 0.01 and ***p < 0.001.

To investigate the pathogenesis of OA, primary chondrocytes were initially treated with pro‐inflammatory cytokine (TNF‐α) to comprise a conventional cell model simulating the destructive environment of progressive OA [30]. Then, RNA‐sequencing analysis of these chondrocytes was performed. Untreated primary chondrocytes were used as controls. The gene ontology (GO) analysis of the functions of differentially expressed genes (DEGs) showed that canonical glycolysis and mitophagy were significantly enriched (Figure 2A). The heatmap demonstrated that the expression of glycolysis‐related genes was elevated; however, the expression of mitophagy‐related genes (LC3B, Pink1 and Fundc1) was decreased after the TNF‐α treatment (Figure 2B). To validate these findings, additional analyses of both IL‐1β‐stimulated chondrocytes from rats and human OA chondrocytes were conducted using datasets from the Gene Expression Omnibus database. The expression of both glycolysis‐related genes (Hk1, Hk2, Pfkm and Pgk1) and mitophagy‐related genes (such as p62, Pink1 and Fundc1) in these chondrocytes was consistent with our findings (Figure 2C). These results indicated that mitophagy was impaired and glycolysis was enhanced in OA chondrocytes.

Quantitative PCR (qPCR) and western blot (WB) analyses demonstrated that the expression of chondrocyte ECM anabolism‐related genes and proteins, such as Sox9, were restrained, whereas ECM catabolism‐related genes and proteins, such as Mmp3 and Mmp13, were significantly promoted after the pro‐inflammatory cytokine (TNF‐α or IL‐1β) treatment (Figure S1 and Figure 2D), confirming the abnormal homeostasis of OA chondrocytes. In particular, analyses of glycolysis‐related genes and proteins, such as Ldha and Hk2 and mitophagy‐related genes and proteins, such as LC3B, Parkin, Pink1 and p62, further confirmed the promotion of glycolysis and impaired mitophagy in OA chondrocytes (Figure S1 and Figure 2D). Subsequently, a surgical model known as destabilisation of the medial meniscus (DMM), the gold standard for studying the onset and development of posttraumatic OA, was established in mice [31]. Safranin O/fast green staining indicated surface abrasions and superficial irregularities in the cartilage (Figure 2E) and Osteoarthritis Research Society International (OARSI) scores were significantly elevated (Figure 2F) in DMM mouse models. Immunohistochemical (IHC) staining of Ldha and LC3B (markers of glycolysis and mitophagy, respectively) revealed that the expression of Ldha was upregulated and that of LC3B was downregulated in mouse OA cartilage (Figure 2E,G,H). These results validated the impaired mitophagy and enhanced glycolysis in DMM mouse cartilage. Cartilage specimens were collected intraoperatively from the tibial plateau of patients with OA. As shown in Figure 2E,I, more damaged cartilage surfaces and higher OARSI scores were observed on the damaged side than on the intact side. IHC staining of Ldha and LC3B showed elevated expression of Ldha and decreased expression of LC3B in patients with OA, which was consistent with the results observed in mouse OA cartilage (Figure 2E,J,K).

Mitochondrial dysfunction induces intracellular ROS production, promotes chondrocyte apoptosis and contributes to cartilage degeneration [32]. Mitophagy is a process that selectively removes damaged or dysfunctional mitochondria through autophagy, playing an important role in OA. Our findings suggest that mitophagy is impaired and glycolysis is significantly enhanced during the progression of OA (Figure 2L). Activating mitophagy and reprogramming glucose metabolism may restore chondrocyte metabolic homeostasis, thus representing a potential therapeutic strategy for cartilage degeneration in OA.

### 2.2 Characterisation of *Chlorella* and Photothermal Ch@P

**FIGURE 3:**
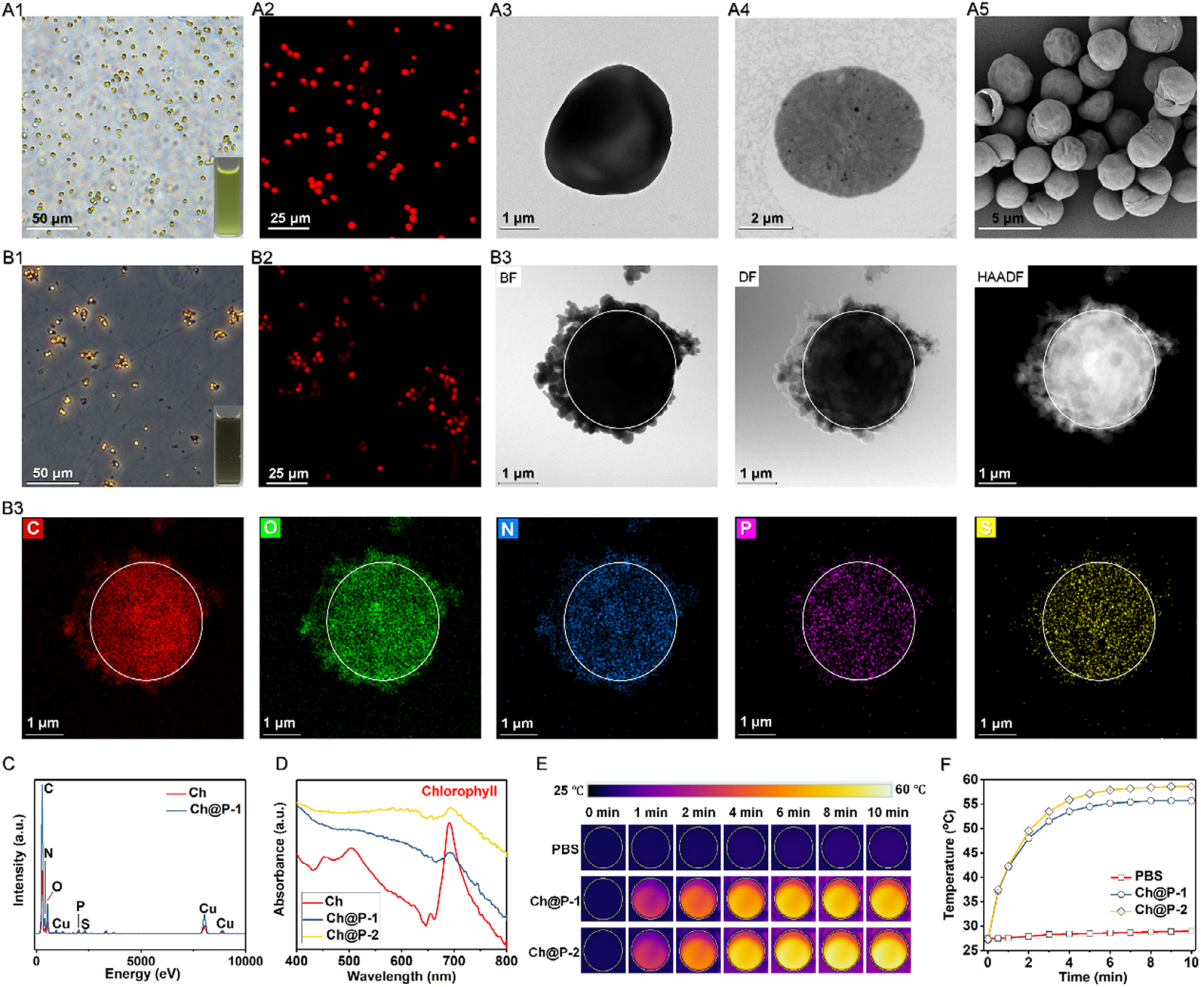
Characterisation of native *Chlorella* and photothermal Ch@P. (A1‐A5) The bright‐field microscopic image (A1), fluorescence image (A2), typical TEM image (A3), TEM image after negative staining (A4) and SEM image (A5) of native *Chlorella* cells (Inset in A1: The optical image of *Chlorella* cells in water). (B1–B3) The bright‐field microscopic image (B1), fluorescence image (B2), typical TEM images and elemental mapping of PDA‐engineered *Chlorella* (Ch@P) cells (Inset in B1: The optical image of Ch@P cells in water). (C) Energy dispersive X‐ray spectroscopy (EDS) of *Chlorella* and Ch@P. (D) UV‐Vis absorption spectroscopy of *Chlorella*, Ch@P‐1 and Ch@P‐2 (Ch@P‐1 and Ch@P‐2 represent the Ch@P prepared with an initial dopamine concentration of 5 mM and 10 mM, respectively). (E and F) Infrared thermal images and corresponding photothermal heating curves of Ch@P‐1 and Ch@P‐2 under irradiation by an 808 nm NIR laser with a power density of 1.0 W/cm^2^.

*Chlorella* is a kind of spherical, unicellular and photosynthetic freshwater algae, with a diameter of 5–10 µm (Figure 3, A1‐A5). It shows good dispersion in water (Figure 3A1). The *Chlorella* shows strong red fluorescence under 350 nm excitation due to the presence of chlorophyll in *Chlorella* cells (Figure 3A2). Transmission electron microscopy (TEM) and scanning electron microscopy (SEM) verify the typical spherical unicellular morphology of native *Chlorella* (Figure 3A3–5). The *Chlorella* was then engineered with PDA to obtain a photothermal PDA‐engineered *Chlorella* biohybrid (denoted as Ch@P) by a dopamine oxidation and self‐polymerisation process (Figure S2) [33]. This dopamine self‐polymerisation process led to the colour change of *Chlorella* suspension from green to dark grey (inset in Figure 3A1,B1). This mild engineering procedure did not change the fluorescence of *Chlorella* cells (Figure 3B2). TEM of Ch@P shows the well‐maintained morphology of *Chlorella* and distinct aggregates of PDA around the *Chlorella* cell. The element mapping indicates the clear and homogeneous distribution of C, O and N elements of PDA on the surface of the *Chlorella* after 2 h self‐polymerisation of dopamine. The C, O and N elements in Ch@P are distinctly increased, as compared to the native *Chlorella* (Figure 3C). The ultraviolet‐visible (UV‐Vis) absorption spectrum of Ch@P indicates that the absorption peak of chlorophyll around 690 nm is well‐preserved after the PDA engineering process (Figure 3D). Besides, the Ch@P shows a distinct increase in optical absorption intensity in the range of 400–600 nm due to the introduction of PDA onto the *Chlorella*, which is consistent with that of PDA NPs (Figure S3) [34]. Moreover, this increase of absorption intensity in the range of 400–600 nm is dependent on the initial dopamine concentration (Here, Ch@P‐1 represents Ch@P with an initial dopamine concentration of 5 mM, while Ch@P‐2 is Ch@P with an initial dopamine concentration of 10 mM). It seems that the increase in initial dopamine concentration led to more significant dopamine self‐polymerisation and more PDA on the Ch@P. To figure out the photothermal conversion capability of Ch@P after the introduction of PDA, the photothermal effect of Ch@P was investigated under 808 nm NIR irradiation. As shown in Figure 3E,F), Ch@P showed distinct photothermal conversion capability, in contrast to the native *Chlorella*. It is noteworthy that the photothermal temperature increases when the initial dopamine concentration increases from 5 to 10 mM. Moreover, the photothermal temperature can be easily controlled by adjusting the power density of the 808 nm NIR laser (Figure 3E,F and Figure S4). The temperature of the Ch@P could reach even more than 60°C by increasing the power density of the NIR laser to 1.5 W/cm^2^ (Figure S4).

### 2.3 Ch@P Alleviates Chondrocyte Extracellular Matrix Degradation

The progressive degeneration of articular cartilage, a central pathological event in OA, is regulated by chondrocyte function. As the resident cell type, chondrocytes orchestrate the synthesis and secretion of the ECM, thereby maintaining cartilage homeostasis [35]. We investigated whether natural *Chlorella* and the artificial *Chlorella* biohybrid of Ch@P were effective in alleviating chondrocyte ECM degradation. Initially, the biocompatibility of *Chlorella* (abbreviated as Ch in Figures) and Ch@P with chondrocytes was investigated. We found that both *Chlorella* and Ch@P showed no significant cytotoxicity or impact on chondrocyte proliferation at given concentrations (Figures S5 and S6). Moreover, treatment with Ch@P decreased the mRNA expression of Hk2 and increased the expression of *Pink1* dose‐dependently, demonstrating activation of mitophagy and suppression of glycolysis in IL‐1β‐stimulated chondrocytes (Figure S7). Thus, the concentration of Ch@P was optimised to 2 × 10^6^ cells/mL for use in subsequent experiments.

**FIGURE 4:**
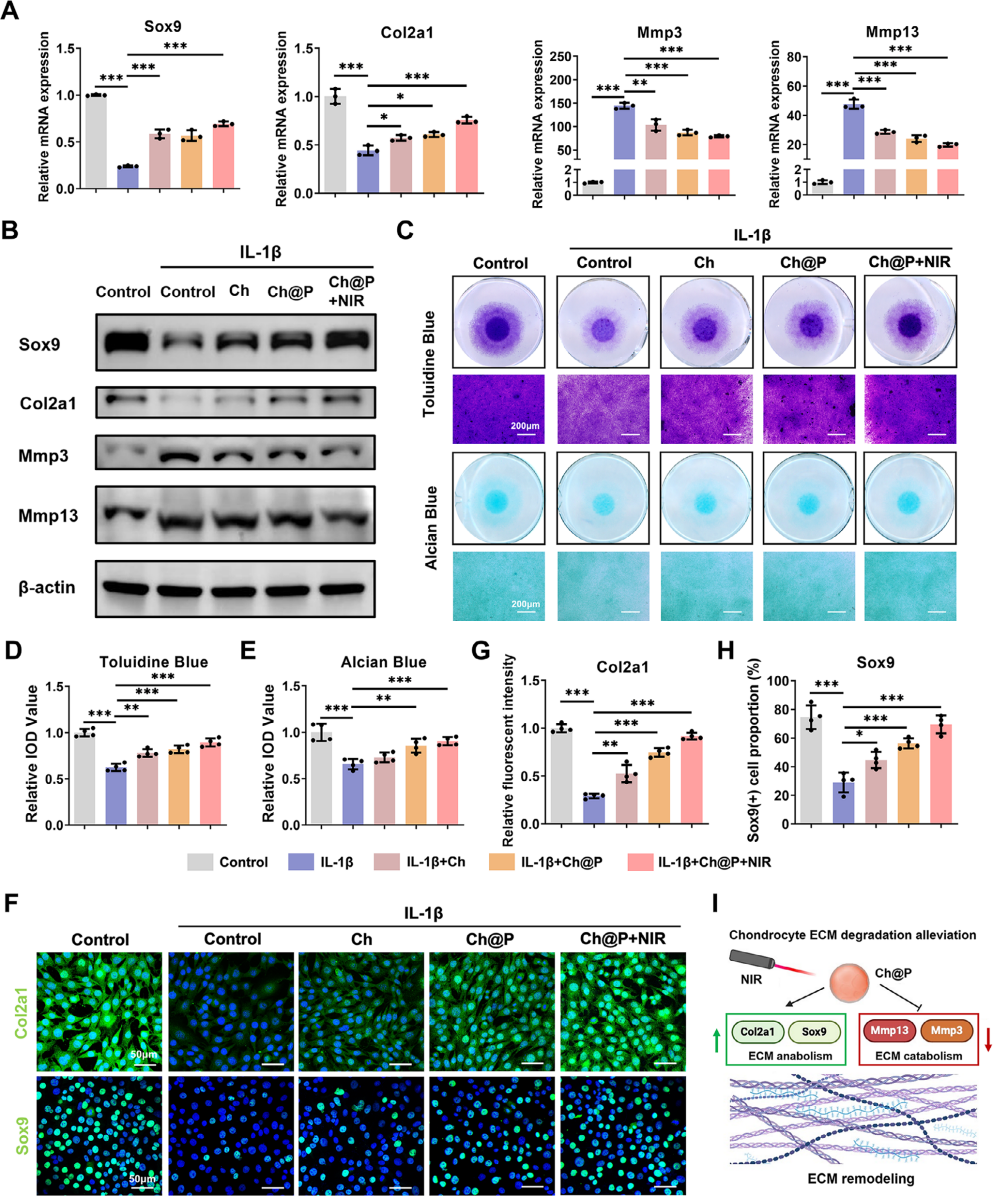
Ch@P alleviates chondrocyte extracellular matrix (ECM) degradation. (A) Quantitative analysis of the mRNA expression of Sox9, Col2a1, Mmp3 and Mmp13 after different treatments. (B) WB of Sox9, Col2a1, Mmp3 and Mmp13 in different groups. (C–E) Alcian blue and toluidine blue staining images and quantitative analyses of chondrocyte micromass cultures after different treatments. (F–H) Immunofluorescence staining and quantitative analyses of Col2a1 and Sox9 in different groups. (I) Illustration of the effect of Ch@P on chondrocyte ECM anabolism and catabolism. Data are presented as means ± SD derived from three or four replicates. *p < 0.05, **p < 0.01 and ***p < 0.001.

Then, the effects of Ch@P on chondrocyte homeostasis and ECM degradation were investigated. The expression of chondrocyte ECM anabolism (Sox9 and Col2a1) and catabolism (Mmp3 and Mmp13) related genes and proteins was evaluated. After the IL‐1β treatment, the mRNA and protein expression levels of Sox9 and Col2a1 decreased, while the expression levels of Mmp3 and Mmp13 increased, representing a chondrocyte degeneration model in vitro (Figure 4A,B and Figure S8). Ch@P reversed mRNA and protein expression levels of ECM anabolic markers (Sox9 and Col2a1) and catabolic markers (Mmp3 and Mmp13). Moreover, this reversal was synergistically enhanced by NIR irradiation (Figure 4A,B and Figure S8). Chondrocyte micromass cultures were used to assess ECM degradation. Alcian blue and toluidine blue staining revealed that Ch@P promoted extracellular collagen matrix preservation in IL‐1β‐stimulated chondrocytes and this promotion was further enhanced by NIR irradiation (Figure 4C–E). In addition, immunofluorescence analyses showed that Ch@P treatment significantly improved the ratio of Col2a1‐ and Sox9‐positive cells, further confirming the alleviation of chondrocyte ECM degradation (Figure 4F–H). These results indicated that Ch@P can facilitate chondrocyte homeostasis and alleviate chondrocyte ECM degradation in the inflammatory OA microenvironment (Figure 4I).

### 2.4 Ch@P Maintains Mitochondrial Homeostasis by Regulating Mitophagy

**FIGURE 5:**
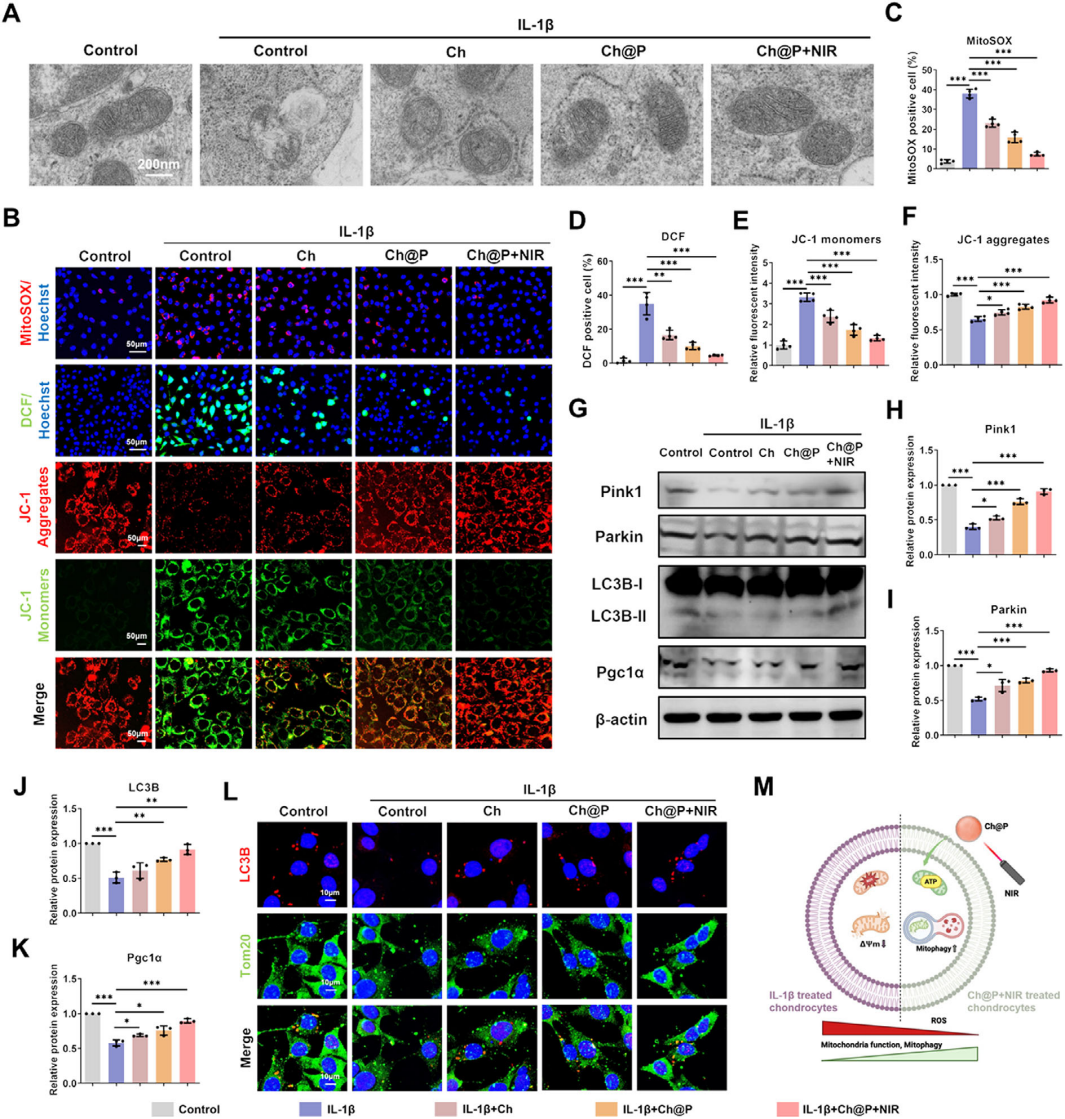
Ch@P maintains mitochondrial homeostasis by regulating mitophagy. (A) TEM images of mitochondria in chondrocytes after different treatments. (B–F) Immunofluorescent staining and quantitative analyses of cytosolic ROS level, mitochondrial ROS level, JC‐1 monomers (green) and JC‐1 aggregates (red) in different groups. (G–K) The protein expression of mitophagy‐related markers (Pink1, Parkin, LC3B and Pgc1α) in different groups. (L) Co‐localisation of LC3B and Tom20 in different groups. (M) The diagram shows that Ch@P maintains mitochondrial homeostasis by activating mitophagy. Data are presented as means ± SD derived from three or four replicates. *p < 0.05, **p < 0.01 and ***p < 0.001.

Mitochondria play key roles in chondrocyte functions through ATP generation and the regulation of apoptosis and autophagy. Mitochondrial dysfunction, which is often accompanied by mitochondrial morphological and structural abnormalities, can contribute to cartilage degeneration in OA [36]. We initially used TEM to assess changes in mitochondrial morphology and structure. As shown in Figure 5A, abnormal mitochondrial structures, including cristae rupture and membrane swelling, were observed in IL‐1β‐treated chondrocytes. In contrast, Ch@P could significantly preserve the morphology and structure of mitochondria. Cytosolic (DCFH‐DA staining) and mitochondrial ROS (MitoSOX staining) levels in chondrocytes were evaluated using confocal laser scanning microscopy. As shown in Figure 5B–D, IL‐1β treatment increased cytosolic ROS and mitochondrial ROS levels, whereas Ch@P decreased the proportion of DCF‐ and MitoSOX‐positive chondrocytes. NIR irradiation further decreased the ROS levels in chondrocytes. Increasing evidence indicates that dysfunctional and depolarised mitochondrial accumulation can increase ROS production and cause an inflammatory response, inducing the synthesis of cytokines and matrix metalloproteinases (MMPs) and contributing to cartilage degeneration in OA [37]. Accordingly, JC‐1, a lipophilic fluorescence probe for mitochondrial membrane potential (∆Ψm), was used to evaluate the mitochondrial membrane integrity. The ratio of intracellular red (JC‐1 aggregates) to green fluorescence (JC‐1 monomers) indicated the depolarisation level of ∆Ψm [38]. As shown in Figure 5B,E,F, Ch@P could markedly increase the ratio of red to green fluorescence, indicating the recovery of ∆Ψm in chondrocytes.

Because mitophagy is impaired in OA chondrocytes, we explored whether Ch@P protects against chondrocyte degradation by regulating mitophagy. The WB analysis showed a significantly decreased protein expression of mitophagy markers (Pink1, Parkin, LC3B and Pgc1α) in IL‐1β‐stimulated chondrocytes (Figure 5G–K), reconfirming the impaired mitophagy in OA chondrocytes. As expected, Ch@P treatment significantly increased the expression of these proteins. This effect was more pronounced under NIR irradiation (Figure 5G–K). Mitophagy involves wrapping mitochondria with autophagosomes to form mitochondrial autophagosomes. Therefore, we used Tom20 and LC3B as mitochondrial and autophagosome markers, respectively, to verify the mitophagy pathway. We found that IL‐1β significantly inhibited the formation of mitochondrial autophagosomes. However, Ch@P treatment notably promoted the recruitment of autophagosomes to the mitochondria, thereby facilitating mitophagy in the presence of IL‐1β (Figure 5L). The Mitochondrial autophagosome is thought to fuse with the lysosome and the mitochondria are transported by the autophagosome into the lysosome. Finally, the mitochondrial content is degraded by lysosomes to synthesise new mitochondria [39]. Immunofluorescence results showed that Ch@P treatment could reverse the IL‐1β‐induced decrease of lysosomes and mitochondrial formation (Figure S9), suggesting the activation of mitophagy. Overall, these results indicated that Ch@P maintained mitochondrial homeostasis by regulating mitophagy (Figure 5M).

### 2.5 Ch@P Alleviates Glycolysis and Reprograms Energy Metabolism in Chondrocytes

**FIGURE 6:**
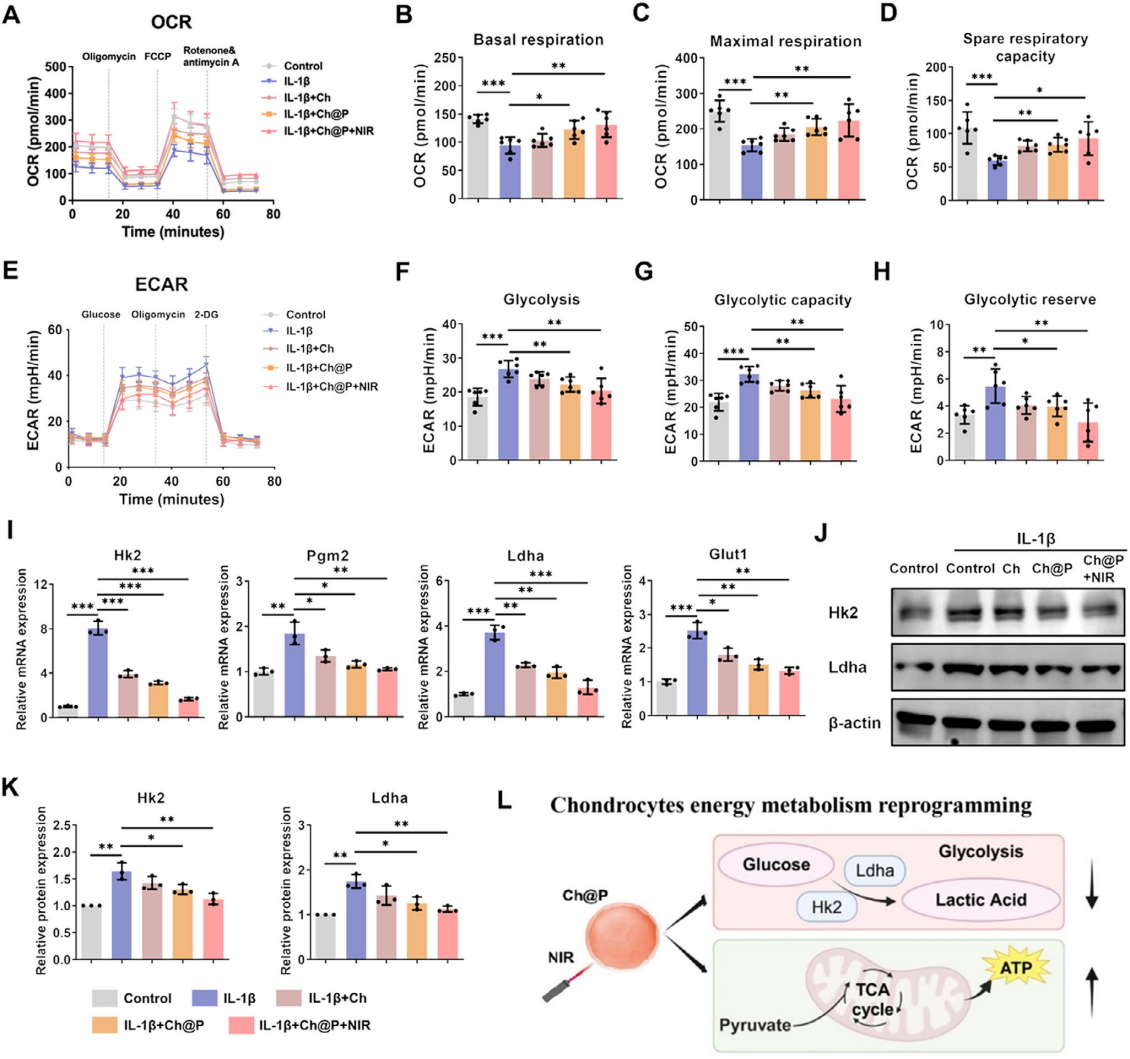
Ch@P alleviates glycolysis and reprograms energy metabolism in chondrocytes. (A) Normalised oxygen consumption rate (OCR) of chondrocytes after different treatments. (B–D) Effects of different treatments on basal respiration, maximal respiration and spare respiratory capacity in OCR assay. (E) Normalised extracellular acidification rate (ECAR) of chondrocytes after different treatments. (F–H) Effects of different treatments on glycolysis, glycolytic capacity and glycolytic reserve in the ECAR assay. (I) The mRNA expression of glycolysis‐related markers in different groups. (J and K) The protein expression and quantitative analyses of glycolysis‐related markers in different groups. (L) The diagram shows that Ch@P alleviates glycolysis and reprograms energy metabolism in chondrocytes. Data are presented as means ± SD derived from three or six replicates. *p < 0.05, **p < 0.01 and ***p < 0.001.

The chondrocyte energy metabolism involves glycolysis, OXPHOS and other metabolic pathways [40]. Inspired by the activity of Ch@P in mitochondrial homeostasis, we investigated its potential effects on chondrocyte energy metabolism. The oxygen consumption rate (OCR) and extracellular acidification rate (ECAR) of chondrocytes were assessed utilising a Seahorse extracellular flux analysis. The reduced OCR, particularly basal and maximal respiration, in IL‐1β‐induced chondrocytes could be substantially rescued by Ch@P with or without the NIR irradiation (Figure 6A–D), suggesting the protective effect on mitochondrial function. Moreover, IL‐1β induced an increase in glycolysis and glycolytic capacity could be prominently suppressed by Ch@P treatment (Figure 6E–H). As shown in Figure 6I, mRNA expression of glycolysis‐related genes (Hk2, Pgm2, Ldha and Glut1) increased under inflammatory conditions, whereas Ch@P downregulated the expression of these genes. In addition, the expression levels of glycolysis‐related proteins (Hk2 and Ldha) decreased after Ch@P treatment (Figure 6J,K). All these results indicated that Ch@P treatment alleviated IL‐1β‐induced glycolysis and reprogrammed the energy metabolism phenotype of chondrocytes (Figure 6L).

### 2.6 Ch@P Remodels Chondrocyte Homeostasis In Vitro via Activating AMPK‐Sirt1 and HSP70 Pathways

**FIGURE 7:**
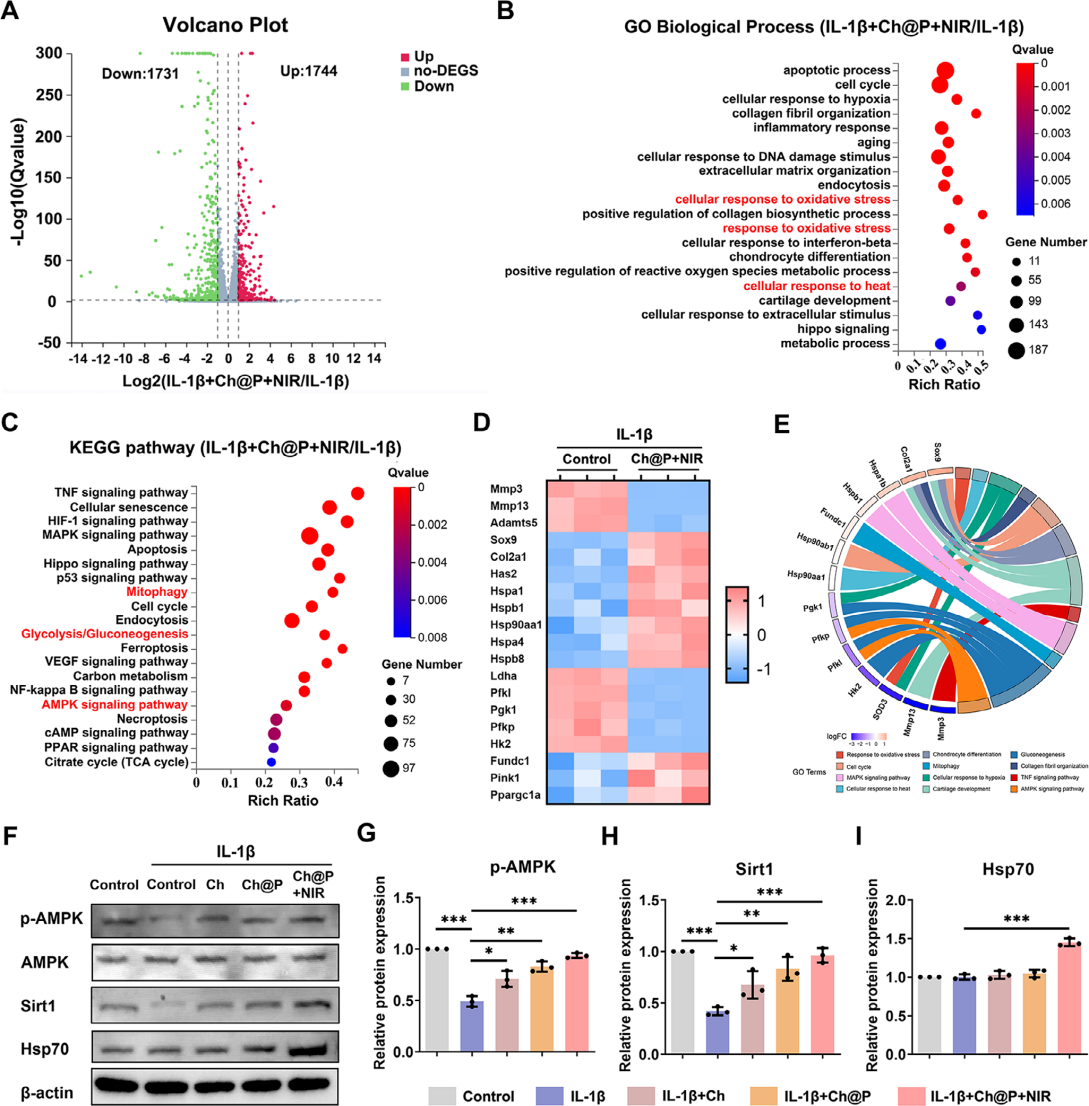
Ch@P + NIR activates AMPK‐Sirt1 and HSP70 pathways synergistically. (A) Volcano plot of DEGs in chondrocytes exposed to IL‐1β, with or without Ch@P + NIR treatment. (B and C) GO and KEGG enrichment analysis of the DEGs. (D) The heatmap of DEGs related to ECM metabolism, mitophagy, glycolysis and response to heat between the two groups. (E) Chord diagram of the correlation between pathways regarding mitophagy, glycolysis, AMPK pathway and relevant DEGs. (F–I) The protein expression of AMPK‐Sirt1 and Hsp70 in different groups. Data are presented as means ± SD derived from three replicates. *p < 0.05, **p < 0.01 and ***p < 0.001.

We then explored the underlying biological mechanisms of Ch@P using RNA sequencing. Given that NIR irradiation plays a synergetic role in promoting the biological activity of Ch@P, IL‐1β‐induced chondrocytes were treated with Ch@P + NIR and the mRNA was extracted for transcriptome sequencing. IL‐1β‐induced PBS‐treated chondrocytes were used as controls. As shown in Figure 7A, the volcano plot identified 3475 DEGs, of which 1744 were upregulated and 1731 were downregulated. GO enrichment analyses of the biological processes revealed that apoptosis, cell cycle, ECM organisation, and response to oxidative stress were significantly enriched (Figure 7B). Notably, the cellular response to heat was also enriched, suggesting a crucial role for the photothermal performance following NIR irradiation. The KEGG pathway enrichment analysis indicated significant enrichment of mitophagy, glycolysis and AMPK signalling pathways (Figure 7C). Changes in the expression of genes related to ECM metabolism, mitophagy, glycolysis and heat response were visualised using a heatmap (Figure 7D). The expression of genes related to ECM catabolism (Mmp3, Mmp13 and Adamts5) and glycolysis (Ldha, Pfkl, Pfkp, Pgk1 and Hk2) was notably inhibited, whereas the expression of genes related to ECM anabolism (Sox9, Col2a1 and Has2) and mitophagy (Fundc1 and Pink1) was promoted after Ch@P + NIR treatment. Notably, the expression of heat‐responsive genes (Hspa1, Hspb1 and Hsp90aa1) increased significantly under NIR irradiation. The chord diagram further illustrates the correlation between pathways related to mitophagy, glycolysis, AMPK and relevant DEGs (Figure 7E).

AMPK, a heterotrimeric complex, is a key molecule involved in the regulation of energy metabolism and mitochondrial homeostasis [41]. AMPK is activated by various stimuli and is necessary for the maintenance of glucose homeostasis. Studies have revealed that the expression of phosphorylated AMPK is significantly decreased in both experimental mouse models and human knee OA chondrocytes [42, 43]. Previous studies have indicated that AMPK activation can reverse impaired mitophagy in OA chondrocytes by regulating the Sirt1 signalling pathway [43]. In this work, we found that Ch@P + NIR treatment can activate AMPK and promote the expression of its downstream protein Sirt1 in IL‐1β‐induced chondrocytes (Figure 7F–H). Hsp70, the most abundant heat shock protein in cells, is preferentially induced in response to cellular stress, protecting cells from injury and promoting the refolding of denatured proteins. Upregulation of Hsp70 can promote chondrogenic gene expression and can participate in Pink1‐mediated mitophagy [44, 45]. The upregulated expression of the Hsp70 protein after Ch@P + NIR treatment confirmed the protective role of Ch@P + NIR (Figure 7F,I). In general, these results indicate that Ch@P + NIR may synergistically regulate mitophagy and glucose metabolism through the AMPK‐Sirt1 pathway and activate Hsp70 in response to the photothermal effect.

### 2.7 Ch@P Alleviates Cartilage Degeneration of OA In Vivo

**FIGURE 8:**
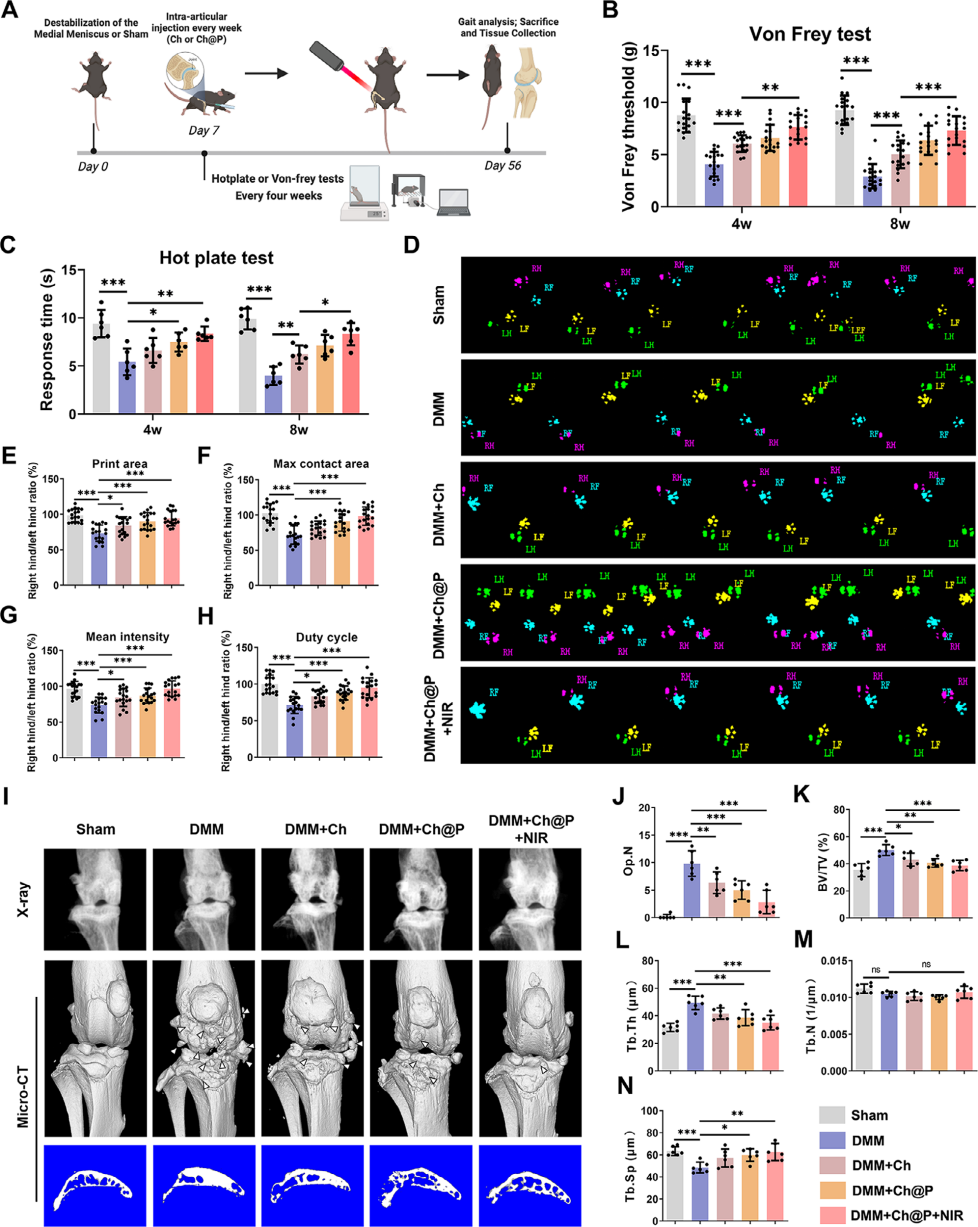
Ch@P alleviates cartilage degeneration of OA in vivo. (A) Illustration of the workflow of in vivo experiments. (B and C) Von‐Frey and hotplate test results at different time points after DMM surgery in different groups. (D–H) Representative images of gait collection (D) and gait analysis including the print area (E), maximum contact area (F), mean intensity (G) and duty cycle (H) of mice in different groups. (I–N) Representative X‐ray and micro‐CT 3D reconstruction images of knee joints and quantitative analyses of osteophyte number (J), BV/TV (K), trabecular thickness (L), trabecular number (M) and trabecular separation (N). Data are presented as means ± SD derived from six or eighteen replicates. *p < 0.05, **p < 0.01 and ***p < 0.001.

The in vivo biosafety evaluation, as indicated by H&E staining of the major organs, showed that both *Chlorella* and Ch@P exhibited favourable biosafety (Figure S10). The photothermal performance of Ch@P in vivo was then detected using an NIR thermal imaging system. Local temperature changes were much higher in the Ch@P group than in the control group (Figure S11), validating the photothermal properties of Ch@P under NIR irradiation. A surgical model of DMM was established in mice and the therapeutic effects of Ch@P on OA progression were investigated in vivo. As illustrated in Figure 8A, *Chlorella* and Ch@P were injected into the right knee joints of mice once a week. For the Ch@P + NIR group, NIR irradiation was performed at an intensity of 1.0 W/cm^2^ for 10 min after the injection of Ch@P. Four and eight weeks post‐surgery, lower limb pain threshold and gait were assessed, respectively. As shown in Figure 8B,C, Ch@P + NIR treatment relieved the severity of lower limb mechanical allodynia and the mice exhibited increased pain tolerance at both the early (4 weeks) and middle‐late (8 weeks) stages following DMM surgery. Gait analysis also indicated an improvement in the abnormal gait in mice after Ch@P + NIR treatment. Moreover, the decreased print area, maximum contact area, mean intensity and duty cycle of the affected limb were significantly improved after Ch@P + NIR treatment (Figure 8D–H). Osteophyte formation and subchondral bone sclerosis are the other major characteristics of OA. Radiographic analyses revealed significant differences in the knee joint structures of the mice in these groups. In contrast to the DMM group, which exhibited prominent osteophyte formation and accelerated subchondral bone ossification, the Ch@P + NIR group showed a significant reversal of these pathologies. The treatment effectively inhibited osteophyte formation and ameliorated subchondral osteosclerosis, as reflected by changed bone microarchitecture metrics (Figure 8I–N).

**FIGURE 9:**
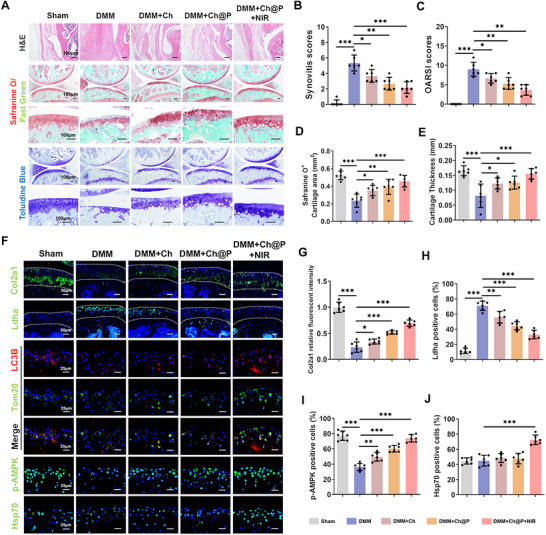
Ch@P + NIR treatment maintains cartilage homeostasis, enhances mitophagy and reduces glycolysis in vivo. (A–E) Representative images of H&E staining, Safranine O/fast green and toluidine blue staining (A) and corresponding quantitative analyses of the synovitis score (B), OARSI score (C), cartilage area (D) and cartilage thickness (E). (F–H) Representative immunofluorescence images of Col2a1, Ldha, LC3B/Tom20 co‐localisation, *p*‐AMPK, Hsp70 and corresponding quantitative analyses. Data are presented as means ± SD derived from six replicates. *p < 0.05, **p < 0.01 and ***p < 0.001.

Elevated OARSI and synovitis scores revealed severe cartilage degeneration and synovial hyperplasia in the DMM‐induced model. The administration of Ch@P + NIR caused a significant decrease in cartilage degradation and synovial hyperplasia (Figure 9A–E). Immunofluorescence staining revealed a significant reduction in the expression of Col2a1 and an increase in the expression of Ldha in the articular cartilage after DMM surgery, whereas these changes were largely reversed by Ch@P + NIR treatment (Figure 9F–H). Immunofluorescence co‐localisation staining of LC3B and Tom20 showed that Ch@P + NIR could rescue the decreased autophagosome recruitment to the mitochondria after DMM surgery (Figure 9F). Moreover, Ch@P + NIR treatment significantly increased the expression of p‐AMPK and Hsp70 (Figure 9F,I,J).

## 3 Discussion

Osteoarthritis (OA) is a chronic degenerative disease that affects the entire joint. Risk factors for OA include age, obesity, inflammation and joint malalignment, which can influence OA progression [21, 46, 47, 48]. Mechanical, inflammatory and metabolic factors are involved in the complex pathogenesis of OA, but the pathogenesis has not been fully elucidated yet. OA seriously affects the quality of life and causes disability in patients [1, 49]. However, the current pharmacological treatments are mostly related to relief of symptoms and there are no disease‐modifying OA drugs [8, 50].

OA is closely related to metabolic diseases. Chondrocytes energy metabolism abnormality exacerbates cartilage degeneration and OA progression. In healthy joints, chondrocytes maintain physiological and metabolic homeostasis; however, they experience a pathological shift in metabolic homeostasis characterised by mitochondrial dysfunction and glucose metabolic disturbances in the OA environment [51, 52]. Our results provide evidence of impaired mitophagy and glucose metabolism disorders in OA chondrocytes in vitro and in vivo (Figure 2). Therefore, targeting chondrocyte energy metabolism may be a potential therapeutic strategy for treating OA [53]. In this study, we revealed that natural *Chlorella* plays a vital role in preventing cartilage degeneration and delaying the progression of OA by restoring mitochondrial function, promoting mitophagy and reprogramming glucose metabolism in chondrocytes (Figure 1).

Mitochondria maintain metabolic homeostasis by providing energy to cells [54]. The structural integrity of the mitochondria is a prerequisite for their biological functions. Mitochondrial morphological and structural abnormalities and depolarisation of ΔΨm often suggest mitochondrial dysfunction [55]. Our results showed that IL‐1β treatment induced cristae rupture, membrane swelling and ΔΨm depolarisation. However, Ch could significantly preserve mitochondrial structure and recover the ∆Ψm (Figure 5). Disruption of the electron transport chain within mitochondria can induce the accumulation of ROS and lead to cell membrane, protein and DNA damage, which is one of the main causes of OA inflammation [56, 57]. Our results demonstrated that cytosolic and mitochondrial ROS levels increased after IL‐1β treatment, whereas Ch decreased the ROS levels in chondrocytes (Figure 5). Mitochondrial dysfunction results in the accumulation of damaged mitochondria in chondrocytes. Mitophagy serves as a key cellular mechanism to preserve mitochondrial homeostasis by selectively identifying and clearing defective mitochondria as cargo for the autophagic machinery [58]. Studies have shown that activation of mitophagy effectively eliminates aggregates of damaged mitochondria and reduces chondrocyte apoptosis and ECM degradation [36, 59, 60, 61]. In the present study, mitophagy was found to be impaired in OA chondrocytes. Ch activated mitophagy to protect chondrocytes from degradation (Figure 5).

We further wonder how Ch exerts its biological functions. Extracellular vesicles (EVs) are rich in bioactive molecules, including proteins, lipids, nucleic acids, amino acids and metabolites, that indirectly regulate the function of receptor cells through the substance exchange and intercellular signalling [62]. We therefore hypothesised that Ch exerted its biological effects through the secretion of exosomes. To verify this hypothesis, we proceeded to extract Ch‐derived exosomes (Ch EVs) (Figure S12) and then evaluated their biological properties in regulating oxidative stress and activating mitophagy. Remarkably, these Ch EVs showed similar oxidative stress suppression and autophagy activation abilities when compared with the live Ch (Figure S13). Consequently, it can be inferred that Ch might exert its therapeutic effects in part through the secretion of exosomes. More exact mechanisms will be further explored in our future studies.

Mitochondria are essential for energy generation using OXPHOS pathways [63]. Damaged mitochondria fail to efficiently produce energy, causing chondrocyte dysfunction and triggering cartilage degeneration. A previous study has shown that the energy metabolism pattern is altered from OXPHOS to glycolysis in patients with OA [24, 64]. Our results demonstrated that Ch reprogrammed energy metabolism as OXPHOS increased and glycolysis decreased in OA chondrocytes (Figure 6). Glycolysis plays a crucial role in the development of OA. The glycolytic pathway is enhanced in OA inflammatory environments, with Hk2, Pkm2, Pfkfb3 and Ldha abnormally expressed in OA chondrocytes [65, 66, 67]. These enzymes and their continuous enzymatic reactions play essential roles in glycolysis and are involved in the pathogenesis of OA. Therefore, targeting glycolysis, particularly glycolytic enzymes, might be a potential therapeutic strategy for the treatment of OA. Our results indicated that Ch treatment alleviatesd IL‐1β‐induced glycolysis and decreasesd the expression of glycolysis‐related genes (Glut1, Hk2 and Ldha) (Figure 6).

Thermotherapy has been widely used to treat OA in clinical settings [68, 69]; it has multiple beneficial effects on joints, including biological heat production, ROS elimination, inflammation reduction and stimulation of cartilage regeneration [70, 71, 72]. Our data indicate that Ch@P exhibits an excellent NIR photothermal response (Figure 3). Ch@P promoted cartilage ECM anabolism and inhibited its catabolism under NIR irradiation (Figure 4). Mitophagy was enhanced and glucose metabolism was reprogrammed after Ch@P + NIR treatment. Our results also demonstrate that Ch@P + NIR treatment effectively protects against cartilage degeneration, relieves pain sensitivity and ameliorates abnormal gait in OA mouse models (Figure 8). Meanwhile, our study has some limitations. First, the long‐term biosafety, biodistribution, clearance and potential immune responses of Ch and Ch@P after intra‐articular injection remain to be thoroughly evaluated. Second, our study demonstrated that Ch might exert its therapeutic effects in part through the secretion of exosomes. However, the exact bioactive cargos within Ch EVs responsible for antioxidant and mitophagy‐inducing effects were not identified. More research will be conducted in our future studies.

In conclusion, our study revealed that natural *Chlorella* plays a vital role in preventing cartilage degeneration and delaying OA progression by promoting mitophagy to maintain mitochondrial and chondrocyte homeostasis. Furthermore, *Chlorella* reprogrammed chondrocyte glucose metabolism by alleviating glycolysis and promoting OXPHOS in chondrocytes. As a proof of concept, we constructed a photothermal Ch@P biohybrid as a promising agent for OA treatment by enhancing chondrocyte mitophagy, reprogramming chondrocyte glucose metabolism and inducing articular thermal stimulation. We found that the Ch@P biohybrid participated in chondrocyte homeostasis by activating Hsp70 and AMPK‐Sirt1 pathways. This study offers new insights into OA treatment by targeting mitophagy and glucose metabolism via a natural microalga‐based biohybrid, providing new ideas for future OA treatments.

## 4 Experimental Section

### 4.1 Preparation of Ch@P

Ch@P was prepared as follows: Typically, *Chlorella vulgaris* (10 mL) was centrifuged and washed twice using sterile PBS. Then, *Chlorella* was dispersed in a dopamine hydrochloride solution (10 mL, 5 or 10 mM in Tris‐HCl, pH 8.0) and slightly stirred (300 rpm) in the dark at room temperature for 2 h. Finally, Ch@P was obtained by centrifugation and repeated washing using sterile PBS. The prepared Ch@P was then dispersed in sterile PBS at 4°C for further use.

### 4.2 NIR Mediated Photothermal Effect of Ch@P

The NIR‐mediated photothermal effects of Ch@P were also investigated. Briefly, different Ch@Ps with initial dopamine concentrations of 5 and 10 mM were dispersed in PBS and irradiated by an 808 nm fibre‐coupled laser system for 10 min at various power densities (0, 1.0 and 1.5 W/cm^2^). *Chlorella* was used as a control. Ch@P‐1 denotes Ch@P with an initial dopamine concentration of 5 mM. Ch@P‐2 denotes Ch@P with an initial dopamine concentration of 10 mM. The temperature was recorded every 60 s using a thermal‐imaging camera.

### 4.3 Characterisation

TEM, high‐resolution TEM, energy dispersive X‐ray spectroscopy (EDS) and corresponding elemental mapping were performed using a Thermo Scientific Talos F200X G2 electron microscope. FESEM images were acquired using a FEI Quanta 400F microscope. The UV‐Vis absorbance spectra were measured using a UV‐3101 Shimadzu spectroscope. Thermal images were recorded using a Fluke thermal imaging camera (Ti480 PRO Infrared Camera). Bright‐field images of cells were observed using a ZEISS fluorescence microscope. Fluorescence images were captured using a confocal microscope (Leica Microsystems, Wetzlar, Germany).

### 4.4 Animal Surgery

All animal experimental procedures were reviewed and approved by the Laboratory Animal Ethics Committee of the Ninth People's Hospital, Shanghai Jiao Tong University School of Medicine (SH9H‐2024‐A1000‐1). Male C57BL/6J mice were purchased from Shanghai Jihui Laboratory Animal Breeding Co. (Shanghai, China) and randomly assigned to different groups. All animals were maintained in an environment with a temperature range of 22–24°C, consistent humidity between 40% and 60% and a 12°h light/dark cycle, with free access to regular food and water. For the DMM‐induced OA model, the medial meniscotibial ligament was transected. Briefly, mice were anaesthetised, and the right knee joint was surgically prepared. An incision was made in the joint capsule to expose and subsequently resect the medial meniscotibial ligament. The wound was then closed by suturing. In the sham group, the surgical procedure was identical except that the ligament was exposed but not cut. Eight weeks after the operation, the knee joints were harvested and fixed in 4% PFA for further evaluation.

### 4.5 Micro‐CT Scanning

Osteophyte formation and subchondral cancellous bone alterations in mouse knee joints were analysed by micro‐CT (Bruker SkyScan 1275, Kontich, Belgium). Following 48‐hour fixation in 4% PFA and a running water rinse, the samples were stored in 70% ethanol. They were then scanned in specialised tubes under the following parameters: 46 kV voltage, 75 µA current and 9 µm resolution. Subsequent 3D reconstruction enabled the quantification of key microstructural parameters, including bone volume fraction (BV/TV), trabecular thickness (Tb.Th), trabecular number (Tb.N) and trabecular separation (Tb.Sp).

### 4.6 Histology and Immunofluorescent Staining

The knee joint specimens were first decalcified in 10% EDTA (Servicebio, Hubei, China). Subsequently, they were processed for paraffin embedding by serial dehydration in ethanol, incubation in xylene and finally embedding. Serial sections (5 µm thick) from the medial compartment were obtained for histological studies. Staining protocols for Safranin O (Servicebio), H&E (Servicebio), toluidine blue, immunohistochemistry and immunofluorescence were performed as per the manufacturers' instructions, using antibodies detailed in Table S2. Finally, sections were imaged with an optical microscope (Olympus, Japan) and a laser scanning confocal microscope (LSCM, TCS SP8, Leica, Germany).

### 4.7 Animal Behavioural Tests

To evaluate mechanical allodynia (von Frey sensitivity), a set of calibrated von Frey filaments was employed (Xinruan Technology, Chengdu, China). The mice were allowed a 15‐min adaptation period on the elevated grid platform before undergoing the von Frey test. A series of calibrated von Frey filaments was used from underneath the hind paw's plantar surface to determine the 50% force‐withdrawal threshold through an iterative approach.

Thermal allodynia was assessed with a hotplate pain meter (Taimeng, Chengdu, China) set at 55°C. The response latency, defined as the time to limb reactions such as paw shaking, licking, or jumping, was recorded. All tests were conducted by an investigator blinded to the animal assignments and experimental groups.

A CatWalk gait analysis system (CatWalk XT, Noldus, Netherlands) was used to assess pain‐induced gait alterations in different treatment groups. Eight weeks after the surgery, the mice were placed on the walkway of the catwalk system. Before walking, each mouse was allowed to adapt to the environment for 10 min. Subsequently, the mouse walked freely from one side to the other and the gait was recorded and analysed using the CatWalk software, including the print area, maximum contact area, mean intensity and duty cycle.

### 4.8 Human Articular Cartilage Tissue Samples

Human osteoarthritic cartilage was obtained from individuals undergoing total knee arthroplasty at the Ninth People's Hospital affiliated with Shanghai Jiao Tong University School of Medicine. With written informed consent from all participants, cylindrical osteochondral cores (5 mm in diameter) that included the entire cartilage layer together with the underlying subchondral bone were excised from either the medial or lateral region of the tibial plateau using a drilling approach. The harvested tissues were immediately immersed in 4% paraformaldehyde for fixation before downstream processing. Ethical approval was granted by the institutional review board (SH9H‐2019‐T190‐2).

### 4.9 Cell Culture

Primary chondrocytes were prepared from the articular cartilage of neonatal mice. After isolating the cartilage from the joint surface, tissues were cut into small fragments and immediately placed into a trypsin solution (Gibco, USA) for 30 min, then transferred to a 0.2% collagenase II solution (Sigma‐Aldrich, USA) and incubated for approximately 6 h at 37°C. The resulting cell suspension was centrifuged, washed thoroughly and seeded into DMEM/F12 medium (HyClone, USA) enriched with 10% fetal bovine serum (FBS) and 1% penicillin–streptomycin. Cultures were maintained in a humidified incubator set to 37°C and 5% CO_2_. ATDC5 cells, obtained from the cell bank of the Chinese Academy of Sciences (Shanghai, China), were expanded in DMEM (HyClone) containing 5% FBS and 1% penicillin–streptomycin.

### 4.10 RNA Sequencing

To investigate transcriptomic changes, total RNA was extracted from different treated primary chondrocytes and subsequently sequenced by BGI Co., Ltd. (Wuhan, China). GO, KEGG and chord diagram analyses were employed to elucidate the biological functions and signalling pathways associated with the DEGs.

### 4.11 Cell Proliferation and Cell Viability

The effect of Ch@P on cell proliferation and viability was assessed using a cell counting kit‐8 (CCK‐8) assay and calcein/PI live/dead staining. For the CCK‐8 assay, cells were seeded in a 96‐well plate (8000 cells/well) and incubated with varying concentrations of Ch and Ch@P. The cells were incubated with 10% CCK‐8 solution (Dojindo, Kumamoto, Japan) for 2 h at 37°C. The absorbance was measured at 450 nm using a microplate reader (TECAN, Mannedorf, Switzerland). For live/dead staining, cells were seeded on glass‐bottom dishes (5 × 10^4^ cells/well) and treated with different concentrations of Ch and Ch@P. Following a 24°h incubation, the cells were treated with Calcein AM/PI working solution (Beyotime, Shanghai, China) for 30 min at 37°C in darkness. Cell viability was assessed by LSCM.

### 4.12 Micromass Culture

To assess ECM deposition, a chondrocyte micromass culture system was established. Briefly, cells were harvested and adjusted to 1.5 × 10^7^ cells/mL. 10 µL droplets were plated, adhered for 2 h and then carefully overlaid with 500 µL medium. The cultures were maintained for 7 days with medium renewal every other day. Following the culture period, the samples were subjected to alcian blue or toluidine blue staining (Solarbio, Beijing, China) and the stained ECM was quantified with ImageJ.

### 4.13 TEM

The chondrocytes were fixed using 2.5% glutaraldehyde and then dissolved in 1% osmium tetroxide. The samples were then dehydrated, infiltrated and embedded in epoxy resin. The resin blocks were then cut into 70 nm ultrathin sections using an ultramicrotome and these sections were retrieved onto copper grids. After staining with 2% uranyl acetate and 0.4% lead citrate, the sections were observed under TEM (Tecnai F20, FEI, USA).

### 4.14 Cytosolic and Mitochondrial ROS Measurements

Cellular ROS level was measured by a DCFH‐DA fluorescent probe (Beyotime). Briefly, cells were washed with PBS twice and incubated with 10 µM DCFH‐DA at 37°C for 20 min in the dark. The photographs were captured by LSCM. Mitochondrial ROS was measured by the MitoSO Red assay kit (Beyotime). Briefly, cells were washed with PBS twice and incubated with 5 µM MitoSO Red at 37°C for 30 min in the dark. The photographs were captured by LSCM.

### 4.15 ∆Ψm Assay

JC‐1 fluorescence was used to evaluate the mitochondrial membrane potential. Cells were stained with a JC‐1 assay kit (Beyotime) based on the manufacturer's instructions. Briefly, cells were washed with PBS twice and incubated with JC‐1 dye at 37°C for 20 min in the dark. The photographs were captured by LSCM.

### 4.16 Seahorse Metabolic Flux Analysis

The chondrocytes were plated on XF‐96‐cell culture plates at 1 × 10^4^/well (Seahorse Bioscience, USA). The cells were then incubated with different treatments for 24 h. The OCR and ECAR were measured using a Seahorse XF‐96 flux analyser (Seahorse Bioscience). For the mitochondrial stress test, cells were treated with 1.5 µM oligomycin, 0.5 µM FCCP and a combination of rotenone/antimycin A (0.5 µM) to obtain OCR values. For the glycolytic stress test, cells were sequentially exposed to 10 mM glucose, 1 µM oligomycin and 50 mM 2‐DG to measure ECAR. The resulting OCR and ECAR profiles were then analysed to derive key parameters of OXPHOS and glycolytic function.

### 4.17 Immunofluorescence

For immunofluorescence, cells were fixed with 4% PFA for 15 min after washing with PBS and permeabilised with 0.1% Triton X‐100 for 30 min. After blocking with 3% BSA for 1 h, the cells were incubated with a primary antibody at 4°C overnight, followed by a corresponding secondary antibody for 1 h at room temperature. Finally, the nuclei were stained using DAPI (Beyotime) for 15 min in the dark. Images were captured using an LSCM.

### 4.18 qPCR

Gene expression analysis was performed by qPCR. Following total RNA extraction using an HP Total RNA Kit (Omega Bio‐Tek, USA) and quantification via a NanoDrop spectrophotometer (Thermo Fisher Scientific, USA), cDNA was synthesised from the RNA template with a qScript kit (Takara, Japan). The qPCR reactions utilised SYBR Premix Ex Taq (Takara), with GAPDH serving as the endogenous control for normalisation. Primer sequences are listed in Table S1.

### 4.19 WB

Cells were washed three times with pre‐cooled PBS and lysed on ice for 30 min using RIPA buffer (Beyotime) containing 1% protease and phosphatase inhibitors (Thermo Fisher Scientific). Following lysis and centrifugation at 12,000 ×g for 15 min at 4°C, the supernatant was collected for protein concentration quantification with a bicinchoninic acid assay kit (Beyotime). The protein samples were then denatured by boiling at 95°C for 15 min with loading buffer. Equal amounts of protein were separated on 4%–20% SDS‐PAGE gels (GenScript, Nanjing, China) and transferred onto PVDF membranes (Merck, USA). The membranes were blocked with 3% BSA (Biofroxx, Germany) for 1 h at room temperature and subsequently incubated with primary antibodies at 4°C overnight. After washing with TBST (Epizyme, Shanghai, China), the membranes were incubated with a secondary antibody for 1 h in the dark with shaking. Protein bands were visualised using an Odyssey dual‐colour infrared laser imaging system (LI‐COR Biosciences, USA). Antibody details are provided in Table S2.

### 4.20 Statistical Analysis

Data from at least three independent biological replicates are expressed as the mean ± standard deviation (SD). Statistical analyses were conducted with GraphPad Prism 8.0, employing an unpaired two‐tailed Student's t‐test for comparisons between two groups, or one‐way ANOVA with Tukey's post‐hoc test for multiple groups. A *p*‐value of less than 0.05 was considered statistically significant.

## Author Contributions

**Yongyun Chang**: investigation, visualisation, writing – original draft, writing – review and editing. **Keyu Kong**: investigation, visualisation, writing – original draft, writing – review and editing. **Minghao Jin**: investigation, visualisation, writing – original draft, writing – review and editing. **Shasha Liu**: investigation, visualisation, writing – review and editing. **Yangzi Yang**: investigation, visualisation, writing – review and editing. **Yingchun Zhu**: investigation, visualisation, writing – review and editing. **Hua Qiao**: investigation, writing – review and editing. **Wenxuan Fan**: investigation, writing – review and editing. **Xinru Wu**: investigation, writing – review and editing. **Jingwei Zhang**: methodology, writing – review and editing. **Yansong Qi**: methodology, writing – review and editing. **Yongsheng Xu**: methodology, writing – review and editing. **Xiang Feng**: methodology, writing – review and editing. **Pingcuo Nima**: methodology, writing – review and editing. **Piting Ji**: methodology, writing – review and editing. **Tariel Mamuladze**: methodology, writing – review and editing. **Andrei A. Korytkin**: methodology, writing – review and editing. **Huiwu Li**: conceptualisation, methodology, project administration, supervision, writing – review and editing. **Jingke Fu**: conceptualisation, methodology, project administration, supervision, writing – review and editing. **Zanjing Zhai**: conceptualisation, methodology, project administration, supervision, writing – review and editing.

## Ethics Statement

All animal experimental procedures were reviewed and approved by the Laboratory Animal Ethics Committee of the Ninth People's Hospital, Shanghai Jiao Tong University School of Medicine (SH9H‐2024‐A1000‐1). The collection of Human OA articular cartilage samples was approved by the Ethics Committee of the Ninth People's Hospital Affiliated to Shanghai Jiao Tong University School of Medicine (SH9H‐2019‐T190‐2).

## Conflicts of Interest

The authors declare no conflicts of interest.

## Supporting information




**Supporting File**: exp270197‐sup‐0001‐SuppMat.docx.

## Data Availability

The data that support the findings of this study are available from the corresponding author upon reasonable request.
