# Supplementary material for: Artificial Chlorella Biohybrids for Alleviation of Cartilage Degeneration in Osteoarthritis
Source: Exploration (Beijing). 2026 Jul 10;6(4):20250125. doi: 10.1002/EXP.20250125 (PMC13394597; doi:10.1002/EXP.20250125)
Supplement: Supplementary file 1 [file EXP2-6-20250125-s001.docx]

Supplementary materials

Artificial *Chlorella* Biohybrids for Alleviation of Cartilage Degeneration in Osteoarthritis

**Figure S1.** The mRNA expression of genes related to ECM, mitophagy, and glycolysis after inflammatory factor treatment. Data are presented as means ± SD derived from three replicates. **P* < 0.05, ***P* < 0.01, and ****P* < 0.001.

**Figure S2.** Illustration of the preparation process of Ch@P.

**Figure S3.** UV-Vis absorption spectrum of the PDA nanoparticles.

**Figure S4.** Photothermal heating curves of Ch@P (Ch@P-1 and Ch@P-2) under irradiation by an 808 nm laser with different power density of 1.0 W cm^-2^ and 1.5 W cm^-2^. Native Chlorella was used as the control.

**Figure S5.** The relative viability of chondrocytes after Ch and Ch@P treatment at different concentrations for 24 h (A) and 48 h (B). Data are presented as means ± SD derived from five replicates.

**Figure S6.** Live/dead assay (A) and quantitative analysis (B, C) of chondrocytes treated with different concentrations of Ch and Ch@P. Data are presented as means ± SD derived from three replicates.

**Figure S7.** The mRNA expression of Hk2 (A) and Pink1 (B) after Ch@P treatment at different concentrations. Data are presented as means ± SD derived from three replicates. **P* < 0.05, ***P* < 0.01, and ****P* < 0.001.

**Figure S8.** The quantitative analysis of the protein expression of Sox9 (A), Col2a1 (B), Mmp3 (C), and Mmp13 (D) in different treatment groups. Data are presented as means ± SD derived from three replicates. **P* < 0.05, ***P* < 0.01, and ****P* < 0.001.

**Figure S9.** The immunofluorescent staining (A) and quantitative analysis (B, C) of mitochondria and lysosomes in different treatment groups. Data are presented as means ± SD derived from three replicates. **P* < 0.05, ***P* < 0.01, and ****P* < 0.001.

**Figure S10.** Representative images of H&E staining of heart, liver, spleen, lung, and kidney tissues from mice after different treatments.

**Figure S11.** Photothermal photographs of different treated mice (PBS, Ch@P). The line chart showed the temperature changes in two groups.

**Figure S12.** The TEM (A) and the nanoparticle tracking analysis (NTA) (B) of the Chlorella-derived exosomes (Ch EVs).

**Figure S13.** Ch EVs regulated oxidative stress and mitophagy in chondrocytes. (A, B) Immunofluorescent staining and quantitative analyses of cytosolic ROS level, mitochondrial ROS level, JC-1 monomers (green), and JC-1 aggregates (red) in different groups. (C) Co-localization of LC3B and Tom20 in different groups. (D, E) The protein expression of mitophagy-related markers (Pink1, Parkin, and Pgc1α) in different groups. Data are presented as means ± SD derived from three replicates. **P* < 0.05, ***P* < 0.01, and ****P* < 0.001.

**Table S1 Primers used in the qRT-PCR assay**

| Genes | Forward (5′-3′) | Reverse (5′-3′) |
| --- | --- | --- |
| Sox9 | CGTGGACATCGGTGAACTGAG | GGTGCTGCTGATGCCGTAAC |
| Col2a1 | GCTACACTCAAGTCACT | TCAATCCAGTAGTCTCC |
| Mmp3 | CCCTGCAACCGTGAAGAAGA | GACAGCATCCACCCTTGAGT |
| Mmp13 | GGAGCCCTGATGTTTCCCAT | GTCTTCATCGCCTGGACCATA |
| Pink1 | TGTAGAGCGTGGTGGCAATGG | AGGCACCGACTCAGGCATCT |
| p62 | GTTCCAGCACAGGCACAGAAGA | CCCACCGACTCCAAGGCTATCT |
| Glut1 | CCAGCAGCAAGACCGATGAACA | TGAGTGTGGTGGATGGGATGGG |
| Hk1 | TACCGTGTCCTTCCTCCTGTCT | CTGGCTCTTAGGCGTTCGTAGG |
| Hk2 | ATGATCGCCTGCTTATTCACG | CGCCTAGAAATCTCCAGAAGGG |
| Ldha | GCTGCTGATCGTCTCCAATCCA | CACTGCTCCTTGTCTGCGTCA |
| Pkm2 | TCCAGTCACTCCACAGACCTCA | GGCATTCAGCACGGCATCCT |
| Pgm2 | TTGGGACCAGGCTGCTGAGT | CTGCTGAGTGGCTGAGACAACA |
| GAPDH | AGGTCGGTGTGAACGGATTTG | TGTAGACCATGTAGTTGAGGTCA |

**Table S2 The detailed information of** **antibodies**

| Antibodies | Source | Identifier |
| --- | --- | --- |
| Sox9 | Abcam | ab185966 |
| Col2a1 | Abcam | ab34712 |
| Mmp3 | Abcam | ab52915 |
| Mmp13 | Abcam | ab39012 |
| Ldha | Abcam | ab52488 |
| Hk2 | Abcam | ab209847 |
| LC3B | Abcam | ab192890 |
| Parkin | Abcam | ab77924 |
| Pink1 | Proteintech | 23274-1-AP |
| p62 | Proteintech | 18420-1-AP |
| Pgc1α | Proteintech | 66369-1-Ig |
| p-AMPK | Affinity | AF3423 |
| AMPK | Affinity | AF6423 |
| Sirt1 | Affinity | DF6033 |
| Hsp70 | Proteintech | 10995-1-AP |
| β-actin | Cell Signaling Technology | # 4967 |
| Anti-rabbit IgG (H+L) | Cell Signaling Technology | #5151 |
